# The role of fructose 1,6-bisphosphate-mediated glycolysis/gluconeogenesis genes in cancer prognosis

**DOI:** 10.18632/aging.204010

**Published:** 2022-04-11

**Authors:** Chien-Hsiu Li, Ming-Hsien Chan, Yu-Chan Chang

**Affiliations:** 1 Genomics Research Center, Academia Sinica, Taipei, Taiwan; 2 Department of Biomedical Imaging and Radiological Sciences, National Yang Ming Chiao Tung University, Taipei, Taiwan

**Keywords:** ALDOA, FBP1, cancer metabolism, prognosis, bioinformatics

## Abstract

Metabolic reprogramming and elevated glycolysis levels are associated with tumor progression. However, despite cancer cells selectively inhibiting or expressing certain metabolic enzymes, it is unclear whether differences in gene profiles influence patient outcomes. Therefore, identifying the differences in enzyme action may facilitate discovery of gene ontology variations to characterize tumors. Fructose-1,6-bisphosphate (F-1,6-BP) is an important intermediate in glucose metabolism, particularly in cancer. Gluconeogenesis and glycolysis require fructose-1,6-bisphosphonates 1 (FBP1) and fructose-bisphosphate aldolase A (ALDOA), which participate in F-1,6-BP conversion. Increased expression of ALDOA and decreased expression of FBP1 are associated with the progression of various forms of cancer in humans. However, the exact molecular mechanism by which ALDOA and FBP1 are involved in the switching of F-1,6-BP is not yet known. As a result of their pancancer pattern, the relationship between ALDOA and FBP1 in patient prognosis is reversed, particularly in lung adenocarcinoma (LUAD) and liver hepatocellular carcinoma (LIHC). Using The Cancer Genome Atlas (TCGA), we observed that FBP1 expression was low in patients with LUAD and LIHC tumors, which was distinct from ALDOA. A similar trend was observed in the analysis of Cancer Cell Line Encyclopedia (CCLE) datasets. By dissecting downstream networks and possible upstream regulators, using ALDOA and FBP1 as the core, we identified common signatures and interaction events regulated by ALDOA and FBP1. Notably, the identified effectors dominated by ALDOA or FBP1 were distributed in opposite patterns and can be considered independent prognostic indicators for patients with LUAD and LIHC. Therefore, uncovering the effectors between ALDOA and FBP1 will lead to novel therapeutic strategies for cancer patients.

## INTRODUCTION

Among the leading causes of death, cancer remains the major disease in humans. Air pollution and chronic hepatitis-mediated chronic inflammation have resulted in lung adenocarcinoma (LUAD) and hepatocellular carcinoma (HCC), a common subtype of liver cancer, becoming the most prevalent cancer classifications in the world, ranking first and sixth, respectively [1]. The reasons that cancer cells are so deadly include a lack of appropriate diagnostic markers, recurrence/relapse events, drug resistance, and treatment difficulty after cancer cells have metastasized [2]. Various regimens have been formulated to combat cancer, such as chemotherapy, radiotherapy, and immunotherapy. However, the heterogeneity of individual patients leads to limited efficacy of many cancer drugs [3]. Therefore, personalized precision medicine needs continuous improvement.

According to previous views, potential causes of heterogeneity are based on somatic mutations, epigenetic alterations, and cell metabolism [4]. Cellular metabolism includes the biosynthesis of carbohydrates, lipids, proteins, and nucleic acids. Once metabolic reprogramming occurs, it is related to the occurrence of many diseases, such as aberrant glycolysis, leading to diabetes and various phenotypes of cancer. There are many enzymes involved in the metabolism of glucose, among which fructose-bisphosphate aldolase A (ALDOA), a glycolytic and gluconeogenic enzyme involved in glucose metabolism, is an important enzyme that converts fructose-1,6 BP into glyceraldehyde 3-phosphate (G3P) and dihydroxyacetone phosphate (DHAP) intermediate products. We and other researchers have found that ALDOA expression is related to the poor prognosis of many cancers, including lung, liver, pancreatic, colorectal, stomach, bladder, renal, and bone sarcomas [5–12]. Manipulation of ALDOA regulates tumor growth and motility [11–15]. Expression of ALDOA is also related to environmental factors, such as oxygen pressure [9, 16]. Recently, ALDOA was found to have nonenzymatic roles in cancer metastasis, drug resistance, and cancer stemness activity by interacting with different proteins [8, 17–19]. These findings have led to ALDOA being identified as an important therapeutic target. In addition to being catalyzed by ALDOA, fructose-1,6-BP is also subjected to the rate-limiting enzyme in gluconeogenesis and converted to fructose 6-phosphate by FBP1. In many cancers, loss of FBP1 is related to poor patient prognosis [20–26]. Downregulating FBP1 expression can promote tumor cell epithelial-mesenchymal transition, proliferation, and resistance to therapeutic efficacy [21, 27–29]. Interestingly, according to current findings, even if ALDOA regulates fructose-1,6 BP levels during glycolysis, activity of its upstream enzyme FBP1 was not consistent. In addition, the function of FBP1 is controversial. In breast cancer, studies have indicated that FBP1 is suppressed by Snail in basal-like breast cancer [30, 31] but contributes to triple-negative breast cancer progression [32]. However, as the upstream and downstream relationship enzymes of fructose-1, 6 BP, the complete relationship between ALDOA and FBP1 and whether FBP1 and ALDOA have related molecules with a common influence need to be investigated in detail.

This study explored the relationship between FBP1 and ALDOA across cancers and identified a significant correlation between LIHC and LUAD. Based on analyzing the prognosis of TCGA patients, we found that FBP1 and ALDOA are related to survival prognosis and that the expression of FBP1 in patients with high ALDOA is lower, exhibiting a significantly increased correlation. This relationship can be applied to LUAD and LIHC clinical populations, and consistent results were observed in cancer cell patterns. In addition, a molecular simulation signature-related analysis showed that the selected molecules are involved in glycolysis and gluconeogenesis and contribute to cell growth, motility, and DNA repair signatures and can be regulated by similar upstream regulators. These results all demonstrate that in LUAD and LIHC, the relationship between FBP1 and ALDOA affects cancer progression by regulating the same molecules. Thus, in addition to being diagnostic markers, these molecules may also regulate FBP1 and ALDOA during cancer progression.

## RESULTS

### Clinical prognosis correlation of ALDOA and FBP1 across cancers

**Figure 1:**
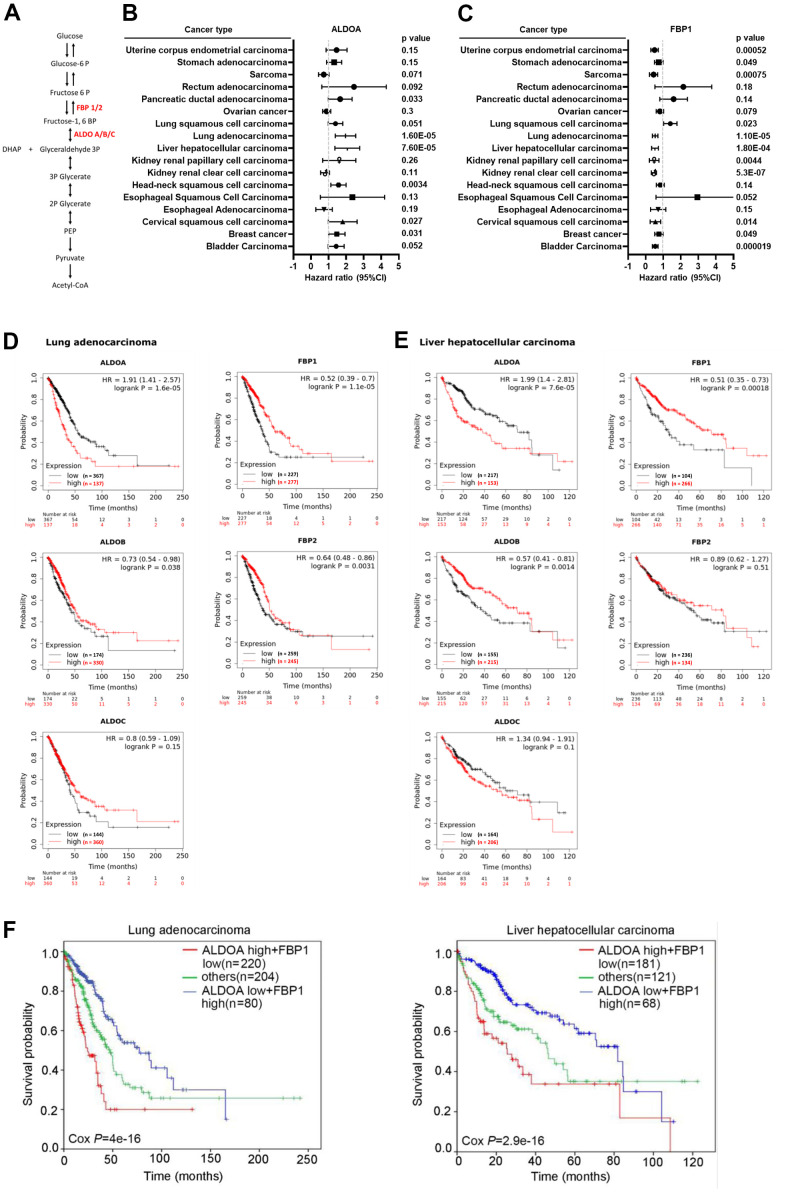
**Significantly opposite trends of ALDOA and FBP1 in LUAD and LIHC.** (**A**) The scheme illustrates the relationship between ALDOA and FBP1 in glycolysis. (**B**) Meta-analysis of the prognostic value of ALDOA from the Kaplan–Meier plotter database. (**C**) Meta-analysis of the prognostic value of FBP1 from the Kaplan–Meier plotter database. (**D**) The association among ALDOA, ALDOB, ALDOC, FBP1, and FBP2 in LUAD from the Kaplan–Meier plotter database. (**E**) The association among ALDOA, ALDOB, ALDOC, FBP1, and FBP2 in LIHC from the Kaplan–Meier plotter database. (**F**) The survival rate correlation between combined ALDOA and FBP1 in LUAD or LIHC from the Kaplan–Meier plotter database. The significance of the differences in (**B**, **C**, **F**) was analyzed using Cox regression. N or n were denoted as sample size. HR was denoted as Hazard ratio.

To investigate the relationship between FBP1 and ALDOA among cancers (Figure 1A), we screened for prognostic value and candidate gene expression across cancers. The related cohort is displayed in Supplementary Figure 1. According to the *p value* and hazard ratio of gene expression in patient overall survival, the results showed that ALDOA is correlated with poor cancer prognosis, including pancreatic ductal adenocarcinoma (*p*=0.033, HR=1.57), LUAD (*p*=0.000016, HR=1.91), LIHC (*p*=0.000076, HR=1.99), head-neck squamous cell carcinoma (*p*=0.0034, HR=1.52), cervical squamous cell carcinoma (*p*=0.027, HR=0.68), and breast cancer (*p*=0.031, HR=1.42) (Figure 1B). In contrast, expression of FBP1 was correlated with better prognosis in uterine corpus endometrial carcinoma (*p*=0.00052, HR=0.48), stomach adenocarcinoma (*p*=0.049, HR=0.72), sarcoma (*p*=0.00075, HR=0.41), lung squamous cell carcinoma (*p*=0.023, HR=1.37), LUAD (*p*=0.000011, HR=0.52), LIHC (*p*=0.00018, HR=0.51), kidney renal papillary cell carcinoma (*p*=0.0044, HR=0.43), kidney renal clear cell carcinoma (*p*=0.00000053, HR=0.45), cervical squamous cell carcinoma (*p*=0.014, HR=0.5), breast cancer (*p*=0.049, HR=0.73), and bladder carcinoma (*p*=0.000019, HR=0.53) (Figure 1C). Between them, FBP1 and ALDOA display significant and opposite trends in LUAD and LIHC. In LUAD and LIHC, high ALDOA expression was greatly correlated with poor patient prognosis (Figure 1D), which was extremely different from the low expression of FBP1 (Figure 1E). The related isoforms of the aldolase family and FBP were also evaluated, and there was no significant correlation between ALDOB/C and FBP2 (Figure 1D, 1E). The combination of ALDOA and FBP1 demonstrated that patients with high ALDOA and low FBP1 had poor prognosis in LUAD and LIHC (Figure 1F).

To verify that this correlation is specific to adenocarcinoma, comprehensive assays were conducted. Interestingly, this opposite trend between ALDOA and FBP1 was also observed in other types of prognosis, such as lung cancer's first progression and post-progression survival (Supplementary Figure 2). Similarly, low FBP1 expression was also related to improved prognosis of lung cancer first progression and post-progression survival, and vice versa, and ALDOA was correlated with poor prognosis. We observed that this correlation was specific to LUAD (Supplementary Figure 2). Similar trends were also observed in LIHC, and the poor prognosis between relapse-free survival, progression-free survival, and disease-specific survival were all correlated with the expression of ALDOA but were opposite to FBP1 (low expression) (Supplementary Figure 3). This result shows that the highly inverse correlation between ALDOA and FBP1 may exist as common diagnostic markers, representing a possible mutual regulatory relationship in LUAD and LIHC.

### Expression of ALDOA and FBP1 in LUAD and LIHC patients

**Figure 2:**
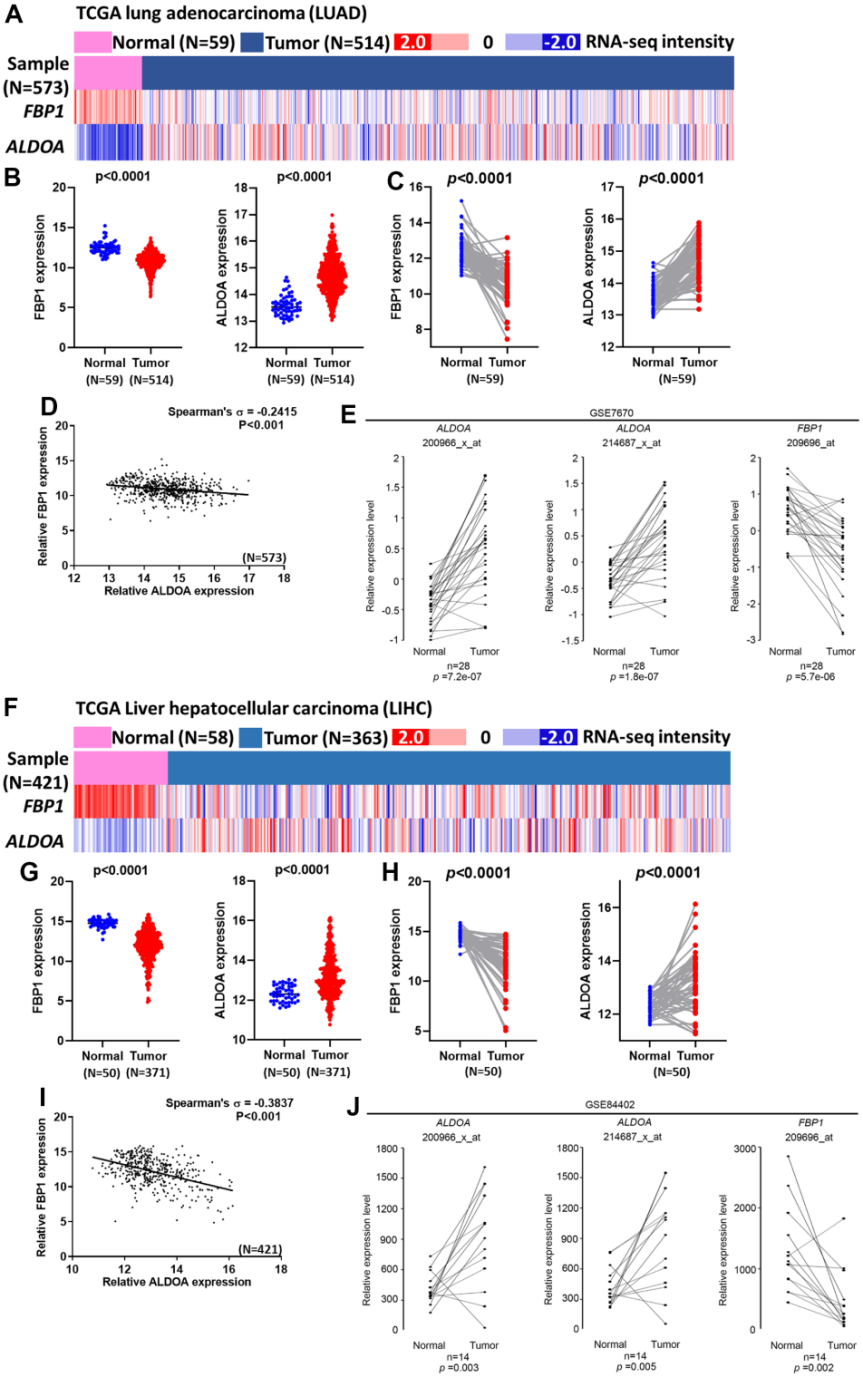
**A negative correlation between ALDOA and FBP1 exists in LUAD and LIHC patients.** (**A**) The heatmap shows the expression of ALDOA and FBP1 in TCGA lung adenocarcinoma (LUAD) patients. (**B**) The related expression of ALDOA and FBP1 in the normal and LUAD tumor groups in TCGA lung adenocarcinoma (LUAD). (**C**) A paired analysis reveals the difference between ALDOA and FBP1 in TCGA lung adenocarcinoma (LUAD). (**D**) The correlation between ALDOA and FBP1 in TCGA lung adenocarcinoma (LUAD). (**E**) A paired analysis revealed the difference between ALDOA and FBP1 in GSE7670. (**F**) The heatmap shows the related expression of ALDOA and FBP1 in TCGA Liver Cancer (LIHC) patients. (**G**) The related expression of ALDOA and FBP1 in normal and LIHC tumor groups in TCGA Liver Cancer (LIHC). (**H**) A paired analysis revealed the difference between ALDOA and FBP1 in TCGA Liver Cancer (LIHC). (**I**) The correlation between ALDOA and FBP1 in TCGA Liver Cancer (LIHC). (**J**) A paired analysis revealed the difference between ALDOA and FBP1 in GSE84402. The significance of the differences in (**B**, **C**, **E**, **G**, **H**, **J**) was analyzed using unpaired Student’s *t*-tests. The significance of the differences in (**D**, **I**) was analyzed using Spearman's rank correlation coefficient. N or n is denoted as sample size.

The role of FBP1 or ALDOA has been reported in multiple cancers [5–12, 20–26]. However, the relationship between ALDOA and FBP1 has not been discussed. To understand the relationship between prognosis and patients, the heatmap results of TCGA analysis are shown in Figure 2A, 2F. In the normal and tumor groups, patients with low FBP1 expression had high ALDOA expression (*p*<0.0001) (Figure 2B, 2G). In the same patient, high ALDOA expression was associated with low FBP1 expression. In contrast, low FBP1 expression was associated with high ALDOA expression (p<0.0001) (Figure 2C, 2H). These data showed that there was a negative correlation between the expression of FBP1 and ALDOA in cancer patients (*p*<0.001) (Figure 2D, 2I). Furthermore, the expression of ALDOA and FBP1 also showed similar trends in multiple cancer stages. We observed a negative correlation between ALDOA and FBP1 from The American Joint Committee on Cancer (AJCC 8th) pathologic stage I to stage III in LUAD or LIHC (Supplementary Figures 4, 5).

Verification of these events was not limited to TCGA datasets. The related clinical datasets were introduced. Identical results demonstrated that an opposite trend between ALDOA and FBP1 was observed between normal and tumor groups. Patients with high ALDOA and low FBP1 expression were observed in the same patients (Figure 2E, 2J). These analyses show that FBP1 and ALDOA have an interplay and correlation in patients with LUAD and LIHC. It also shows that they can be used as indicators of staging and prognostic markers, especially during early stages.

### Expression of ALDOA and FBP1 in LUAD and LIHC cells

**Figure 3:**
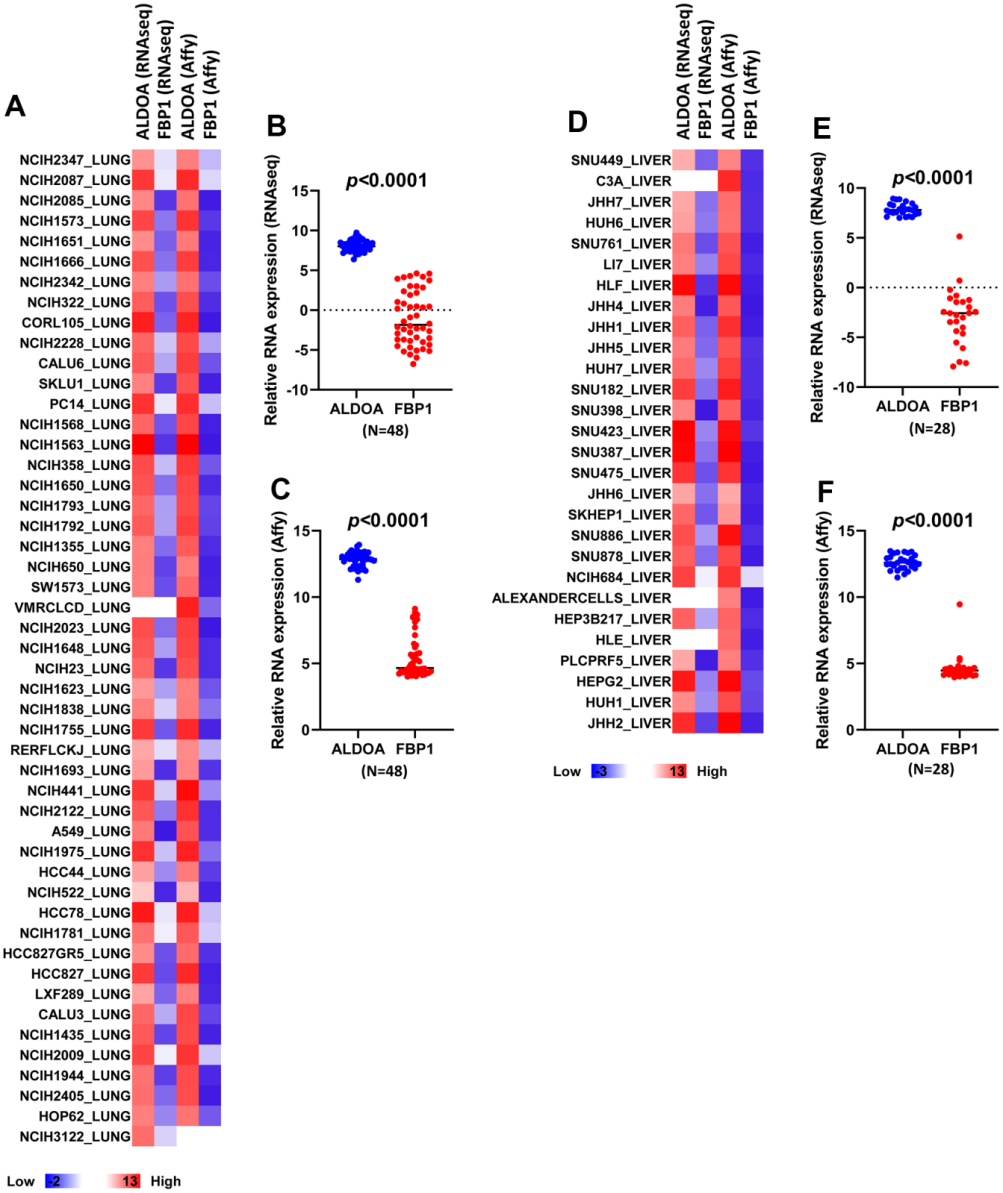
**LUAD and LIHC tumor cells exhibit higher ALDOA expression than FBP1.** (**A**) A heatmap of the related expression of ALDOA and FBP1 in LUAD RNA sequencing data and Affymetrix microarray data. (**B**) Significant differences in ALDOA and FBP1 in LUAD RNA sequencing data. (**C**) Significant differences in ALDOA and FBP1 in LUAD Affymetrix microarray data. (**D**) A heatmap of the related expression of ALDOA and FBP1 in LIHC RNA sequencing data or Affymetrix microarray data. (**E**) Significant differences in ALDOA and FBP1 in LIHC RNA sequencing data. (**F**) Significant differences in ALDOA and FBP1 in LIHC Affymetrix microarray data. The significance of the differences was analyzed using unpaired Student’s *t*-tests. Affy was denoted as Affymetrix. RNAseq is denoted as RNA sequencing. N or n is denoted as sample size.

The Cancer Cell Line Encyclopedia (CCLE) database was established for profiling the expression of specific genes. To understand whether the relationship between ALDOA and FBP1 in patients was also reflected in cell lines, we conducted correlation analysis using the CCLE dataset, which is a complete analysis of the gene expression differences between multiple cancer cell lines. We compared the difference between Affymetrix and RNAseq according to the current derivative stable cell lines that represent LUAD and LIHC cell lines. A heatmap revealed a similar trend between ALDOA and FBP1 in Affymetrix and RNAseq, with low FBP1 expression and high ALDOA expression in both LUAD and LIHC cell lines, both in the AFFY and RNAseq analyses (Figure 3A, 3D). Comparable results showed that the related expression of ALDOA and FBP1 exhibited a significant difference (Figure 3B, 3C, 3E, 3F). Overall, the inverse correlation between ALDOA and FBP1 was consistent in LUAD and LIHC cancer cell lines, supporting the clinical results that FBP1 and ALDOA have an interplay and correlation.

### Molecular signatures involved in FBP1 and ALDOA

**Figure 4:**
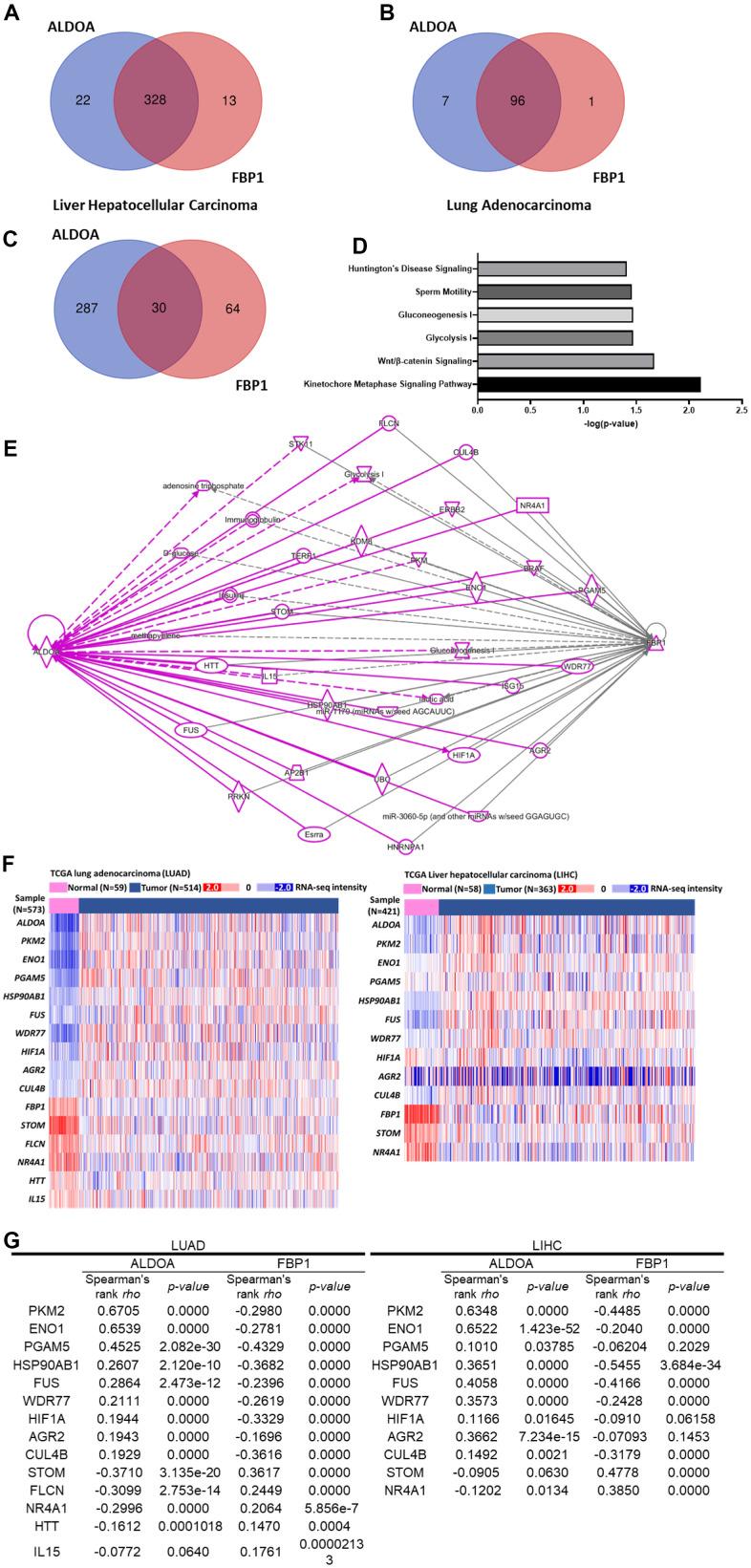
**A gene set analysis shows the relationship between various molecules and ALDOA and FBP1 regulation.** (**A**) Venn diagrams gather molecules related to ALDOA and FBP1 in LIHC. (**B**) Venn diagrams gather molecules related to ALDOA and FBP1 in LIAD. (**C**) The related Venn diagrams gather molecules related to ALDOA or FBP1 in LUAD or LIHC. (**D**) Gene ontology analysis predicts the biological functions of 30 genes. (**E**) An IPA linking molecules that interact between ALDOA and FBP1. (**F**) The heatmap shows the expression of downstream effectors related to ALDOA and FBP1 in TCGA LUAD/LIHC patients. (**G**) Spearman's correlation was used to rank the selected downstream effectors between ALDOA and FBP1 in LUAD/LIHC. The significance of the differences was analyzed using Xena Functional Genomics Explorer website. N or n is denoted as sample size.

Based on the relationship between FBP1 and ALDOA in patients and the related cellular distribution, we hypothesized that ALDOA and FBP1 coregulate specific and identical molecules to control cancer progression. To explore the molecular signature involved in FBP1 and ALDOA, the related molecules ALDOA or FBP1 were selected based on the TCGA database (PanCancer Atlas dataset and Firehose Legacy dataset). Molecules whose correlation with ALDOA or FBP1 exceeded ±0.3 Spearman's correlation were analyzed by Venn diagram (Supplementary Figures 6A, 6B, 7A, 7B) (Supplementary Tables 1, 2, 4, 5). Finally, the identical molecules between these two datasets were analyzed using Venn diagram again, and approximately 328 molecules were identified in LUAD, and 96 molecules were identified in LIHC (Supplementary Figures 6C, 7C) (Supplementary Tables 3, 6). These molecules may correlate with ALDOA and FBP1 expression in LIHC or LUAD (Figure 4A and Supplementary Figure 7A–7C) (Figure 4B and Supplementary Figure 6A–6C). Then, these molecules were subjected to ingenuity pathway analysis (IPA) to identify their involvement in gene ontology-related analysis. In LUAD, the 96 selected molecules played an essential role in those physiological functions by IPA analysis. The results demonstrated that they were primarily involved in cell proliferation, DNA repair, and multiple metabolism pathways. These pathways show a possible relationship with the disease (Supplementary Figure 6D, 6E). To verify that our analysis was correct, according to the current understanding of ALDOA and FBP1, the intersected molecules in LUAD showed that MCM2, NCPAG, PGAM5 and SLC2A1 are related to ALDOA (Supplementary Figure 6F), and TRIM28 is associated with FBP1 (Supplementary Figure 6G). A similar analysis was also conducted in LIHC, and 328 selected genes were analyzed by IPA to profile their possible related signaling pathways (Supplementary Figure 7D). Supplementary Figure 7E shows the signaling pathways that they may participate in, which is consistent with LUAD, primarily cell proliferation and is also related to remodeling of epithelial adherens junctions and multiple cancer signaling pathways as well as the possible signaling interaction relationship. Among these molecules, NCPAG and SLC2A1 are related to ALDOA (Supplementary Figure 7F), and PKM, SMARCA4, and TRIM28 are related to FBP1 (Supplementary Figure 7G). Similar gene ontology results between LUAD and LIHC indicate that similar molecules may be involved. The Venn diagram results identified 30 molecules related to ALDOA and FBP1 in both LUAD and LIHC (Figure 4C) (Supplementary Table 7). Notably, the IPA results not only showed involvement in glycolysis and gluconeogenesis but also showed that they were related to the kinetochore metaphase signaling pathway, Wnt/β-catenin signaling, sperm motility and Huntington's disease signaling (Figure 4D). According to the molecular interaction network of IPA, several molecules are coregulated by ALDOA and FBP1 (Figure 4E). To demonstrate that these molecules may correlate with ALDOA and FBP1 in LUAD or LIHC, the heatmap results of TCGA analysis are displayed in Figure 4F. The correlation results indicated that in LUAD and LIHC, PKM2, ENO1, PGAM5, HSP90AB1, FUS, WDR77, HIF1A, AGR2, and CUL4B were positively correlated with ALDOA and negatively correlated with FBP1. Conversely, STOM and NR4A1 were negatively correlated with ALDOA but positively correlated with FBP1. In addition, FLCN, HTT, and IL15 were negatively correlated with LUAD only, which may be caused by different genetic backgrounds (Figure 4G). Together with these results, all evidence supports that ALDOA and FBP1 coregulate identical downstream effectors to contribute to LUAD and LIHC progression.

### Transcription factors involved in FBP1 and ALDOA

**Figure 5:**
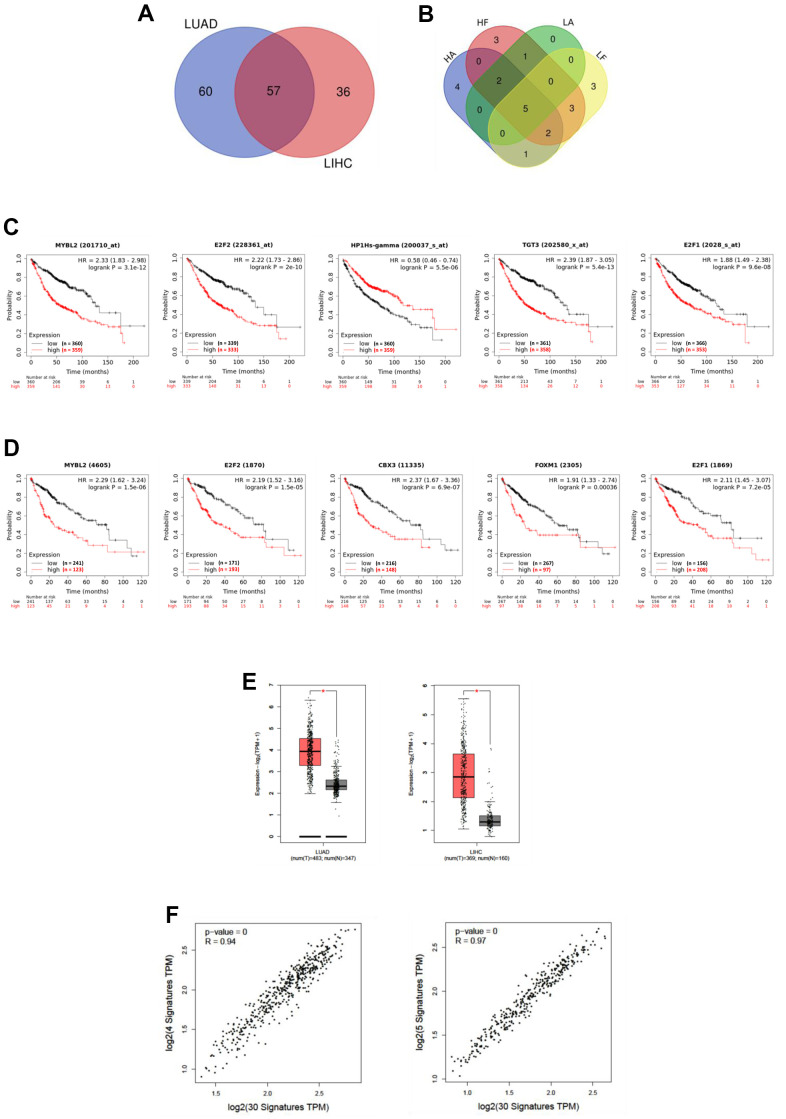
**Upstream regulators are involved in ALDOA and FBP1 regulation.** (**A**) Venn diagrams showing the upstream regulators of ALDOA and FBP1 between LUAD and LIHC. (**B**) Venn diagrams gather upstream regulators with Spearman's correlation values over ±0.3 for ALDOA and FBP1. (**C**) The prognosis of upstream regulators in LUAD. (**D**) The prognosis of upstream regulators in LIHC. (**E**) The upstream regulators selected between ALDOA and FBP1 are increased in LUAD and LIHC. (**F**) The correlation between upstream regulators and downstream effectors of ALDOA and FBP1. The significance of the differences was analyzed using the GEPIA website. HA is denoted as the LIHC PanCancer Atlas dataset. HF is denoted as the LIHC Firehose Legacy dataset. LA is denoted as LUAD PanCancer Atlas dataset. LF is denoted as LUAD Firehose Legacy dataset. N is denoted the normal sample size. T is denoted as the tumor sample size. HR is denoted as hazard ratio.

To unveil the possible transcription factors involved in the regulation of these molecules and their correlation with FBP1 and ALDOA regulation between LUAD and LIHC, we conducted IPA and compared the potential upstream regulators. Approximately 57 upstream regulators were identified in LUAD and LIHC (Figure 5A) (Supplementary Table 8). Furthermore, the correlation value of these transcription factors between ALDOA and FBP1 was evaluated. To rank the most significant upstream regulators, a correlation value over ±0.3 (Spearman's correlation) was selected, and the Venn diagram highlighted approximately five transcription factors, including MYBL2, E2F2, CBX3, FOXM1, and E2F1, that were correlated with ALDOA or FBP1 in both LUAD and LIHC (Figure 5B) (Supplementary Table 9). In addition, the related overall survival rate was computed and significantly correlated with LUAD (Figure 5C) and LIHC (Figure 5D). Notably, the box plot results showed that these upstream regulators may represent gene signature markers for prediction in LUAD or LIHC (Figure 5E). Additionally, we compared the relationship between these transcription factors and the selected molecules in Figure 4C. The results showed that upstream regulators were positively correlated with their downstream effectors in LUAD and LIHC (Figure 5F). These clinical correlation data support that ALDOA and FBP1 coregulate identical downstream molecules via specific upstream regulators in LUAD and LIHC.

## DISCUSSION

The use of targeted inhibitors and immunotherapy has enabled us to progress in combating cancer, but patient differences have led to inconsistent results [33–35]. Metabolic differences and reprogramming may be part of the reason for these inconsistencies [36–38]. Through long-term treatment with targeted inhibitors, it has been demonstrated that cells produce increased lactate, which stimulates connective tissue extracellular matrix, including cancer-associated fibroblasts, to secrete hepatocyte growth factors that promote tumor resistance to the drug [39]. Hence, understanding metabolism in cancer progression is paramount to successful treatment. According to Kaplan–Meier plots generated from pancancer data, ALDOA and FBP1 exert opposite effects on overall survival in LUAD and LIHC (Figure 1) and on LUAD progression and post-progression survival (Supplementary Figure 2) and in LIHC progression-free survival and relapse-free survival (Supplementary Figure 3). In a study that followed previous studies [20–26], we found a strong association between high FBP1 expression and good prognosis in several cancers, such as lung, liver, kidney, and breast cancer (Figure 1C) [22, 30–32, 40–42]. Additionally, high FBP1 correlates with lung squamous cell carcinoma, and low FBP1 correlates with uterine corpus endometrial cancer (p=0.00052). Similar trends were observed in LUAD and LIHC with respect to ALDOA expression and overall survival, first progression, and postp-rogression survival (Supplementary Figures 2, 3), which is consistent with overexpression of ALDOA being associated with poor prognosis in surgical specimens [7, 15, 17, 43]. We previously observed that ALDOA expression correlated more strongly with poor prognosis in lung cancer [9, 10, 18]. In this study, a pancancer analysis found that ALDOA was not only associated with LUAD but was also related to multiple cancers, in agreement with previous reports [5–12] (Figure 1B). Furthermore, TCGA dataset analysis also showed decreased ALDOA and FBP1 expression in individual patients with LUAD and LIHC, suggesting that patients with low FBP1 also exhibit high ALDOA expression (Figure 2D, 2H). In this study, ALDOA and FBP1 exhibited an inverse correlation with patient prognosis, especially in LUAD and LIHC (Figure 1 and Supplementary Figures 1–3). According to the analysis of TCGA datasets, patients with high ALDOA expression had low FBP1 expression that was correlated with various parameters (Figure 2 and Supplementary Figures 4, 5), and the CCLE dataset also consistently reflected these differences (Figure 3). Of note, a negative correlation was observed from stage I to stage III, indicating that expression of ALDOA and FBP1 may play a role in tumor progression, especially during the early stages (Supplementary Figures 4, 5). These results are consistent with previous studies showing that high ALDOA or low FBP1 is associated with the progression of LUAD or LIHC [7, 9, 10, 14, 22, 24, 26, 41, 44–46]. For the first time, we demonstrated that ALDOA and FBP1 correlate with disease progression, especially in LUAD and LIHC.

The inverse expression of ALDOA and FBP1 in LUAD clinical patient tissues can be reflected in their biological functions [44, 47]. Overexpression of FBP1 in lung cancer cells decreases glucose uptake, consequently decreasing lactate production [26]. Interestingly, under hypoxic conditions, HIF-1α increases ALDOA expression, leading to increased lactate production and reducing the degradation of HIF-1α, subsequently promoting the invasive abilities of tumor cells [9]. As a result, cancer cells may increase cellular glycolysis and promote lactate production to modulate the tumor microenvironment by inhibiting the expression of FBP1. Currently, in LUAD or LIHC, transcript levels of FBP1 are understood to be regulated by hypermethylation of its promoter [48] and detransactivation by specific transcription factors, such as ZEB1 [26]. The simulated analysis indicated that the molecules involved in HIF-1α signaling included MMP1, MMP10, MMP12, PKM, PRKCD, RALA, RAN, SLC2A1, and EGLN3 (Supplementary Figures 6D, 7D). In uterine leiomyoma, SLC2A1 and ALDOA were identified as HIF-1α-responsive genes [49]. Nonetheless, it is unclear how they are involved in the regulation of ALDOA and FBP1.

Beyond the clinical and cellular analyses, which indicated that a number of molecules may be involved in ALDOA and FBP1 regulation, the TCGA datasets identified correlations to ALDOA and FBP1 values over ±0.3 (Spearman's rho), which were used to identify 96 molecules in LUAD (Supplementary Figure 6C) and 328 molecules in LIHC (Supplementary Figure 7C), with approximately 30 molecules in common between LUAD and LIHC related to ALDOA and FBP1 (Figure 4C) as well as being increased in LUAD or LIHC tumor groups (Figure 4G and Supplementary Figures 6H, 7H). It is encouraging to find that the Gene Ontology analysis revealed that these 30 molecules are involved in glycolysis and gluconeogenesis, suggesting that the screening method was valid (Figure 4D). Furthermore, Gene Set Enrichment Analysis supports that not only glycolysis and gluconeogenesis but also the kinetochore metaphase signaling pathway, Wnt//β-catenin signaling, sperm motility, and Huntington's disease signaling may play a role in regulating ALDOA and FBP1 (Figure 4D). In addition to glycolysis and glucose synthesis, these identified molecules may also participate in other signaling pathways, including DNA replication, the cell cycle, and cell damage (Figure 4 and Supplementary Figures 6, 7). ALDOA/FBP1 may correlate with LUAD and LIHC, as shown by the network and standard features revealed using an online tool (Figure 4E, 4F and Supplementary Figures 6F, 6G, 7F, 7G). As a prognostic model for overall survival, the first progression survival (Figure 4G and Supplementary Figures 6H, 7H) shows that these molecules potentiate a mutual regulatory relationship and may be important for tumor progression. The molecular interaction analysis identified novel molecules involved in ALDOA or FBP1 regulation, including MSM2, NCAPG, PGAM5, SLC2A1, PKM, SMARCA4 and TRIM2 (Figure 4E, 4F and Supplementary Figures 6F, 6G 7F, 7G), indicating not only the accuracy of the analysis but also identifying novel molecules involved in ALDOA or FBP1 regulation. Unexpectedly, the correlation analysis between the upstream regulator's analysis identified MYBL2, E2F2, CBX3, FOXM1, and E2F1 (Figure 5B), which were significantly elevated in the LUAD or LIHC tumor groups (Figure 5E), with a strongly correlated value (Figure 5F) with utility as biomarkers (Figure 5C, 5D). Based on the fact that knockdown of CBX3 results in an upregulation of FBP1 in pancreatic cells [50] and upregulation of FOXM1 results in a decrease in FBP1 expression in lung cancer [51], it seems the simulation is indeed correct. Nevertheless, the current research is based only on a simulation of the relationship between ALDOA and FBP1 to examine the relationship between ALDOA and FBP1, and further experiments will be necessary to verify the validity this the model. The derivative questions include other proteins that can regulate the activities of FBP1 and ALDOA in the conversion of F-1,6-BP. Overall, this study investigated the interaction of novel molecules involved with ALDOA and FBP1 to determine whether these connections may be related to LUAD and LIHC development. Targeting the ALDOA/FBP1 axis, it may be expected that the molecular regulation and reversal of glycolytic flux will reduce tumor malignancy.

## MATERIALS AND METHODS

### Clinical prognosis correlation profiles of tumor patients

The gene expression in clinical patients with LUAD and LIHC and the related survival correlations, including overall survival, first progression, post regulation survival, relapse-free survival, progression-free survival and disease-specific survival, were analyzed using the Kaplan–Meier Plotter website (https://kmplot.com/analysis/).

### The distribution of genes in LUAD and LIHC patients

Expression of ALDOA, FBP1 and genes in individual patients of TCGA Lung Adenocarcinoma and TCGA Liver Cancer and the distribution in different pathologic stage-related information were downloaded from the Xena Functional Genomics Explorer (http://xenabrowser.net/).

### Gene correlation in LUAD or LIHC patients

Genes that correlated with ALDOA or FBP1 in LUAD or LIHC were downloaded from the TCGA, PanCancer Atlas and TCGA, Firehose Legacy datasets (https://www.cancer.gov/about-nci/organization/ccg/research/structural-genomics/tcga). Spearman's correlation values over ±0.3 were selected from cBioPortal (https://www.cbioportal.org/) for analysis. Furthermore, the gene signature correlations in tumors or normal tissues were analyzed from the GEPIA2 website (http://gepia2.cancer-pku.cn/#index).

### Expression of ALDOA and FBP1 in the CCLE database

The related expression of ALDOA and FBP1 in LUAD (n=49) or LIHC (n=28) human tumor cell lines was analyzed from the Broad Institute Cancer Cell Line Encyclopedia (CCLE) database (https://sites.broadinstitute.org/ccle/).

### Molecule interactions and upstream regulator simulation

Genes selected from the TCGA, PanCancer Atlas and TCGA, Firehose Legacy datasets and correlation values over ±0.3 (Spearman's correlation) were subjected to ingenuity pathway analysis (https://analysis.ingenuity.com) to identify the related relationship between ALDOA or FBP1 in their molecular interactions, gene ontology, and canonical signaling based on the -log(p-value) over 1.52.

### Definition of ALDOA and FBP1 expression levels in patient specimens

Patient specimens (GSE7670 and GSE84402) were downloaded from the Gene Expression Omnibus-NCBI database (https://www.ncbi.nlm.nih.gov/geo/).

### Statistical analysis

The related statistical analyses were conducted using GraphPad Prism 8 (https://www.graphpad.com/scientific-software/prism/) or Excel to derive the unpaired Student’s t-tests. The statistical significance between difference groups was represented by *t*-test at *, p < 0.05; p < 0.01; ***, p < 0.001. A Cox regression analysis was used to define the correlation of survival rate between ALDOA and FBP1 in Lung adenocarcinoma or Liver hepatocellular carcinoma by R software (R package version 4.0.1) (https://www.jstatsoft.org/article/view/v084i02).

## Supplementary Material

Supplementary Figures

Supplementary Table 1

Supplementary Table 2

Supplementary Table 3

Supplementary Table 4

Supplementary Table 5

Supplementary Table 6

Supplementary Tables 7 and 8

Supplementary Table 9
