# Supplementary material for: The role of fructose 1,6-bisphosphate-mediated glycolysis/gluconeogenesis genes in cancer prognosis
Source: Aging (Albany NY). 2022 Apr 11;14(7):3233–58. doi: 10.18632/aging.204010 (PMC9037270; doi:10.18632/aging.204010)
Supplement: Supplementary Figures [file aging-14-204010-s001.pdf]

SUPPLEMENTARY FIGURES

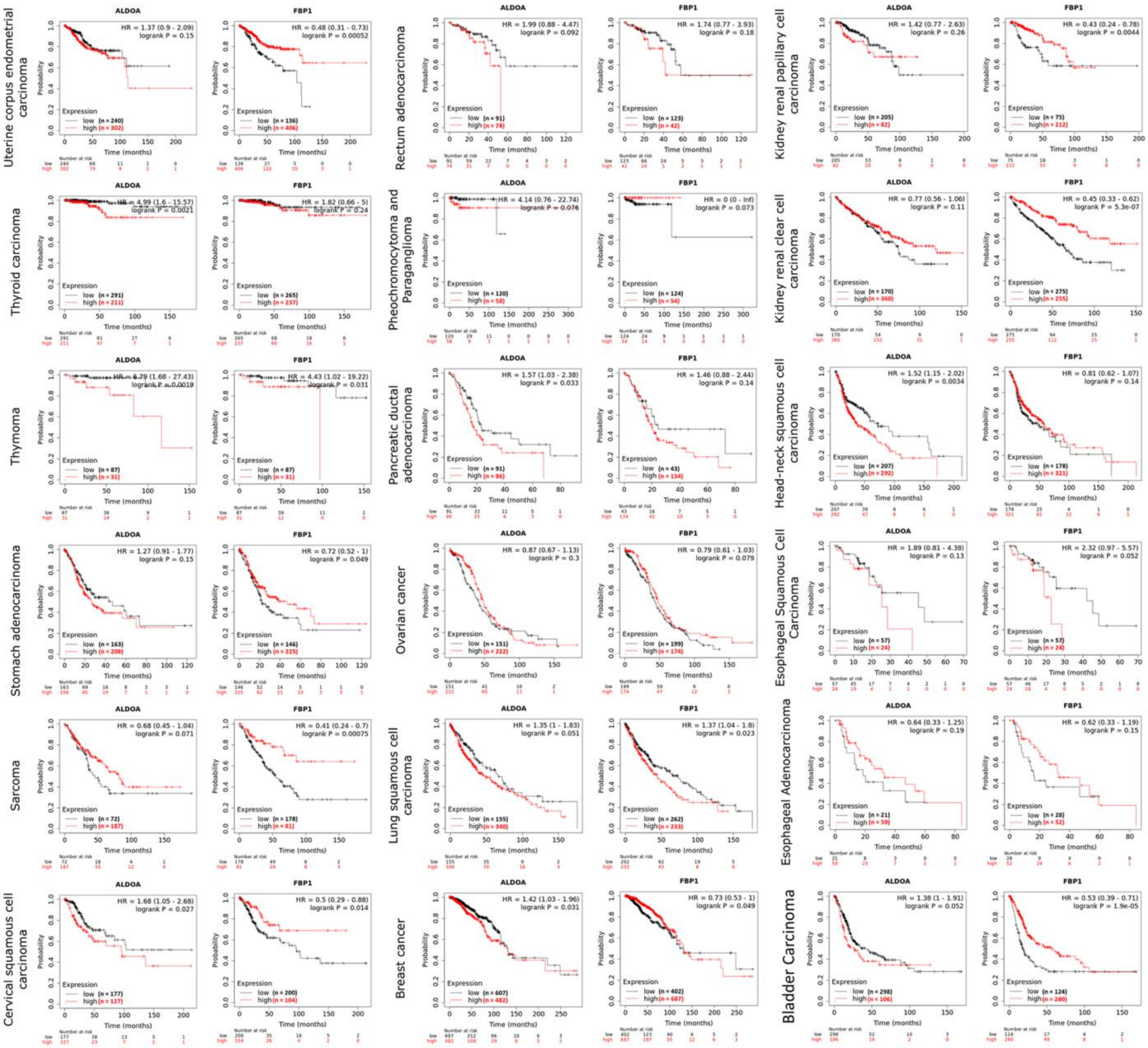

Supplementary Figure 1. The relationship of overall survival between ALDOA and FBP1 across cancers. The overall survival relationship between ALDOA and FBP1 was downloaded from the Kaplan–Meier Plotter website. The HRs, p values, and number of patients are displayed. HR is denoted as hazard ratio. N or n is denoted as sample size.

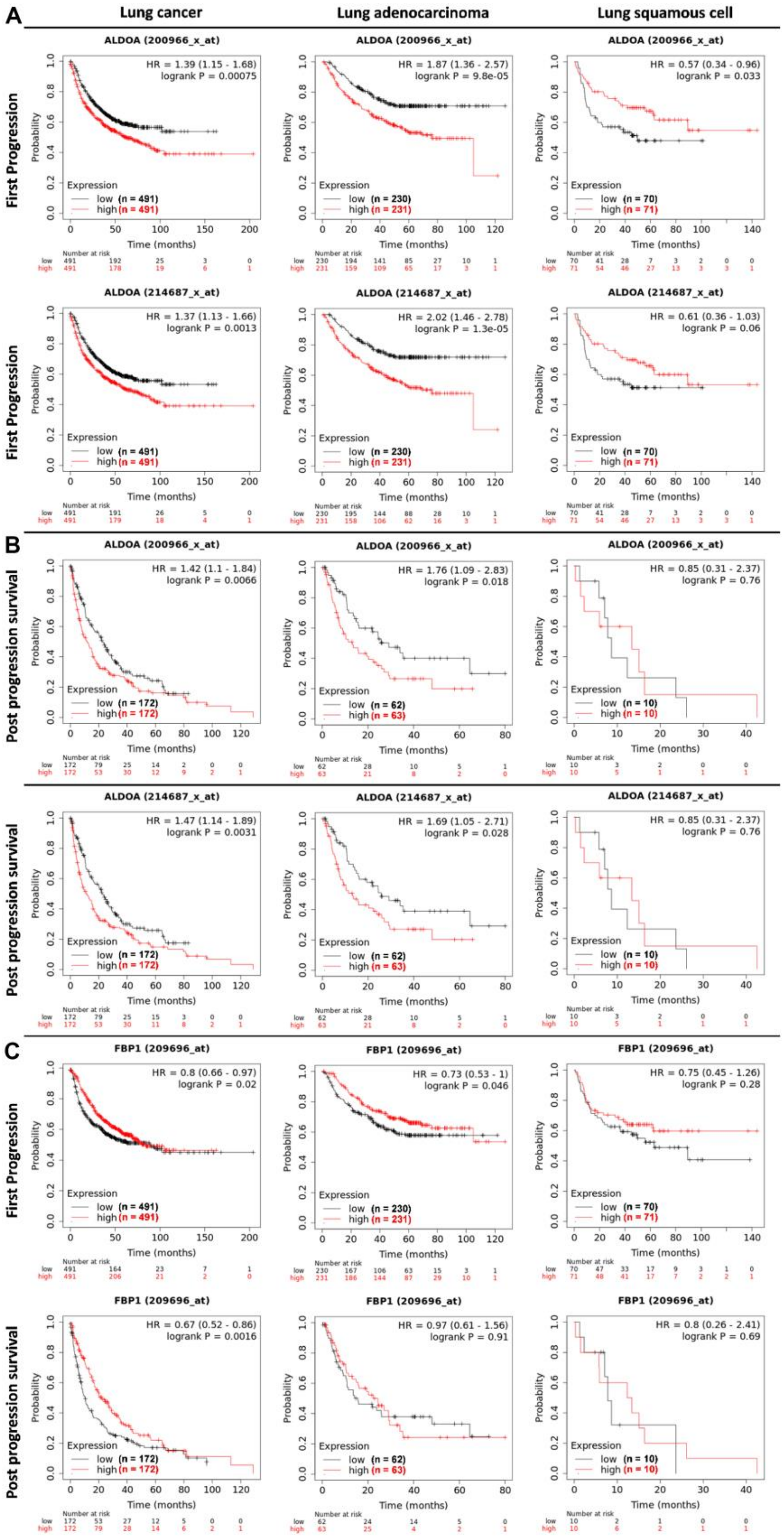

Supplementary Figure 2. Compared to FBP1, ALDOA is correlated with poor prognosis in LUAD. The survival rate correlation between ALDOA or FBP1 in LUAD using the Kaplan–Meier plotter database. (**A**) The first progression prognosis of ALDOA in lung cancer. (**B**) The post progression survival prognosis of ALDOA in lung cancer. (**C**) The first progression or post progression survival prognosis of FBP1 in lung cancer. HR is denoted as Hazard ratio. N or n is denoted as sample size.

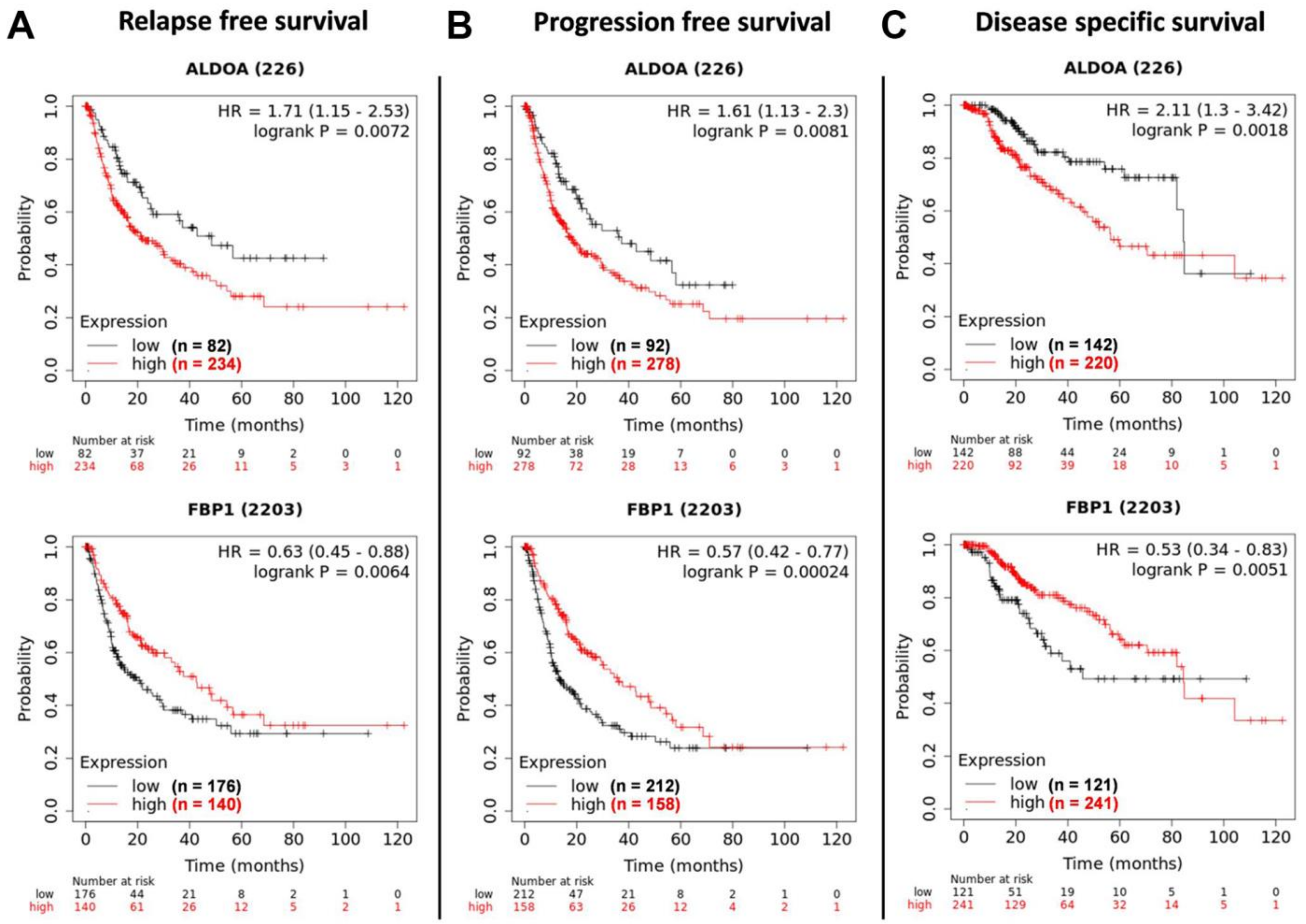

Supplementary Figure 3. High ALDOA and low FBP1 are correlated with poor prognosis of LIHC. Kaplan–Meier plotter datasets reveal the correlation with relapse-free survival in LIHC. (**A**), progression free survival (**B**) and disease-specific survival (**C**) between ALDOA and FBP1 in LIHC. HR is denoted as Hazard ratio. N or n is denoted as sample size.

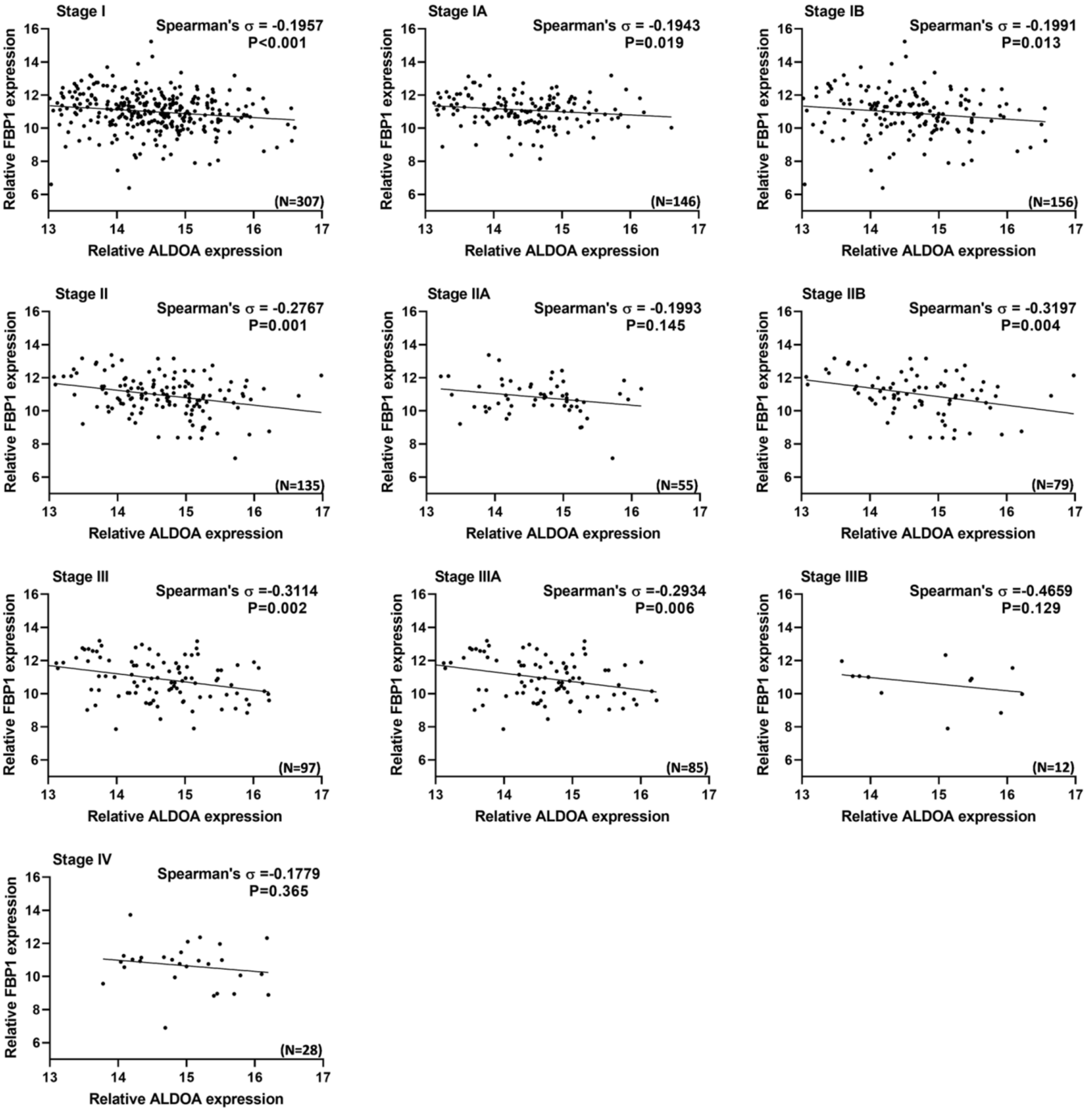

Supplementary Figure 4. ALDOA and FBP1 are negatively correlated in different LUAD stages. The related expression values of ALDOA and FBP1 in LUAD were analyzed from TCGA datasets. Data were downloaded from the Xena Functional Genomics Explorer website, and statistical analyses were performed based on the ALDOA and FBP1 presented by their pathological stage of LUAD. The significance of the differences was analyzed using Spearman's rank correlation coefficient.

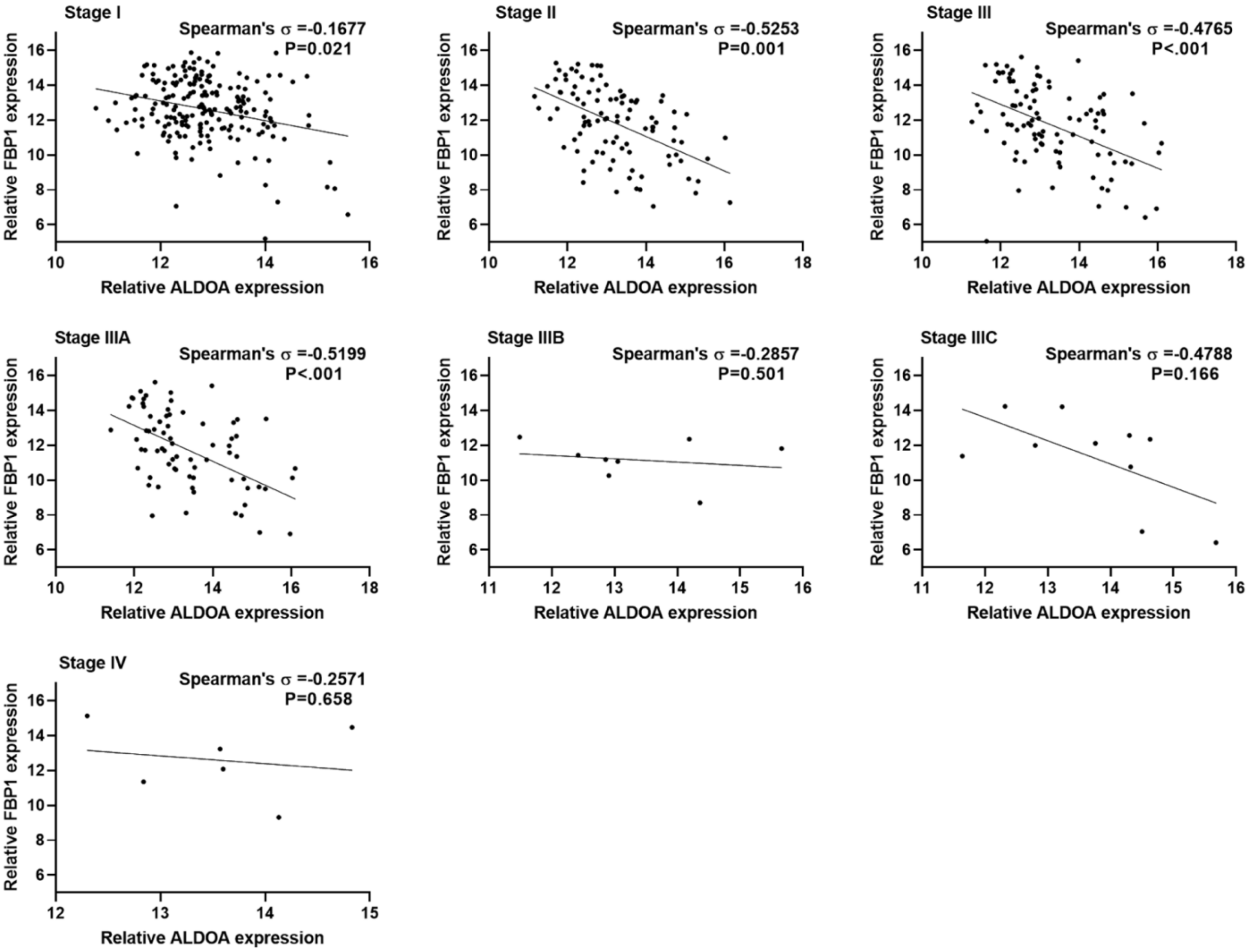

Supplementary Figure 5. ALDOA and FBP1 are inversely correlated in LIHC. The correlation of ALDOA and FBP1 between LIHC stages was generated from TCGA datasets. Data were downloaded from the Xena Functional Genomics Explorer website, and statistical analyses were performed based on the ALDOA and FBP1 presented by their pathological stage of LIHC. The significance of the differences was analyzed using Spearman's rank correlation coefficient.

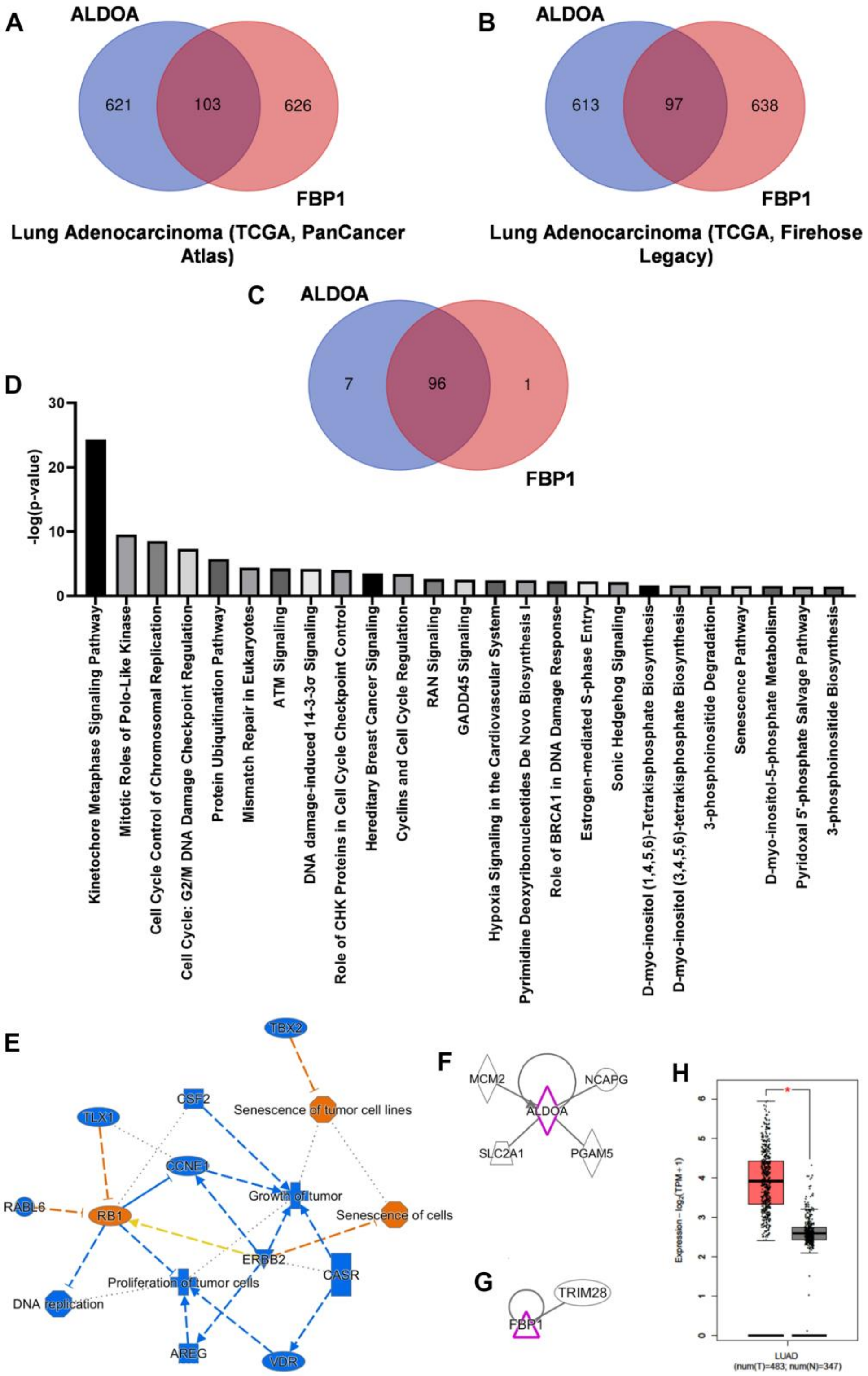

Supplementary Figure 6. The molecular interactions involved in ALDOA and FBP1 in LUAD. (**A**) A Venn diagram gathers molecules of ALDOA and FBP1 in TCGA, PanCancer Atlas. (**B**) A Venn diagram gathers molecules of ALDOA and FBP1 in TCGA, Firehose Lagacy. (**C**) The molecules correlated to ALDOA and FBP1 in TCGA, PanCancer Atlas or FBP1 and TCGA. Firehose Legacy were selected by Venn diagrams. (**D**) The involvement of 96 genes in canonical pathways. (**E**) Possible molecule interaction of 96 genes in gene ontology functions. (**F**) The molecular interactions with ALDOA. (**G**) The molecular interactions with FBP1. (**H**) The molecules selected between ALDOA and FBP1 are increased in LUAD. N is denoted the normal sample size. T is denoted as the tumor sample size. HR is denoted as hazard ratio. The significance of the differences was analyzed using the GEPIA website.

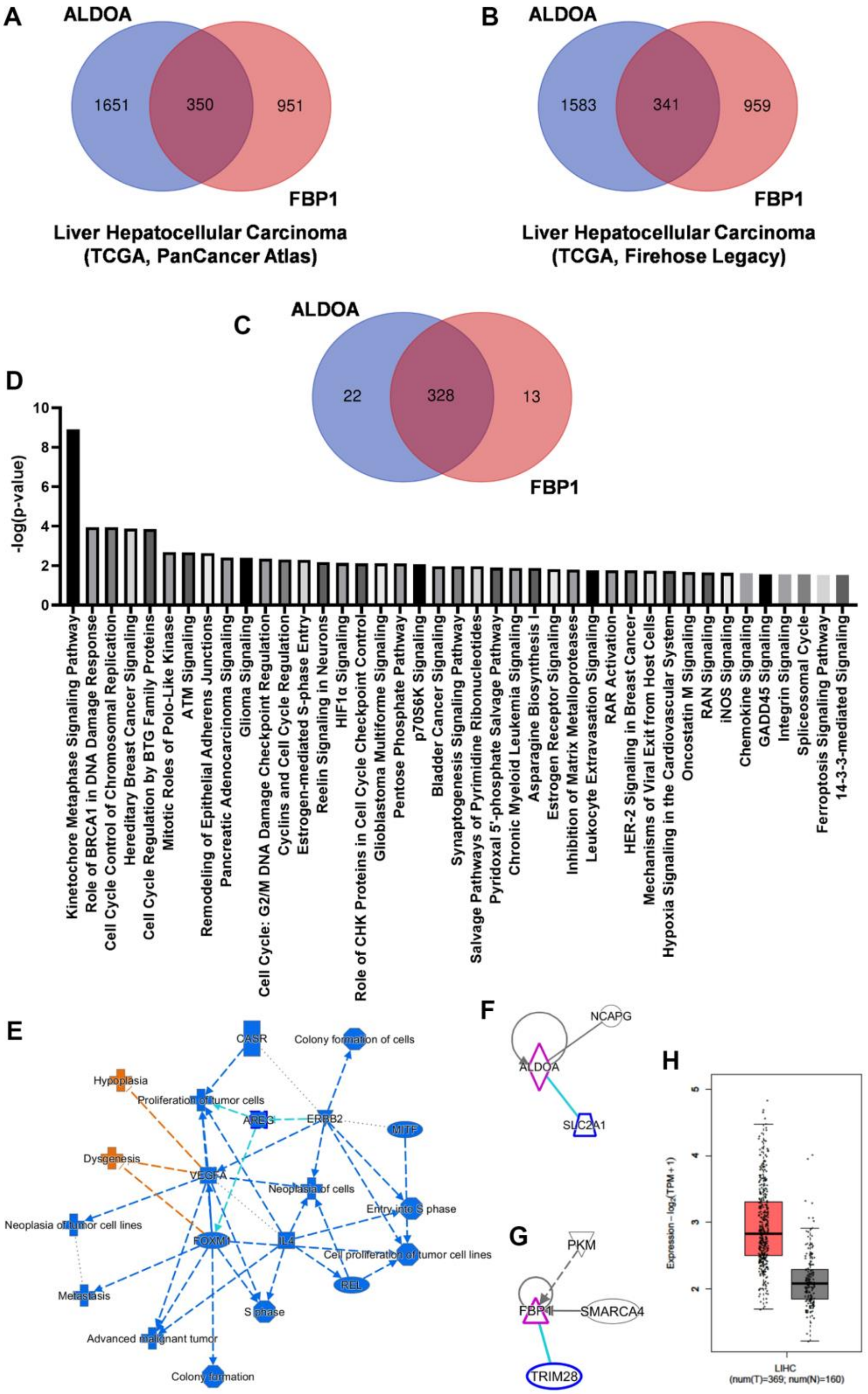

Supplementary Figure 7. The molecular interactions involved in ALDOA and FBP1 in LIHC. (**A**) A Venn diagram gathers molecules of ALDOA and FBP1 in TCGA, and PanCancer Atlas. (**B**) A Venn diagram gathers molecules of ALDOA and FBP1 in TCGA and Firehose Legacy. (**C**) The molecules correlated with ALDOA and FBP1 in TCGA, PanCancer Atlas or FBP1 and TCGA and Firehose Legacy were selected by Venn diagrams. (**D**) The involvement of 328 genes in canonical pathways. (**E**) Possible molecule interaction of 328 genes in gene ontology functions. (**F**) The molecule interaction between ALDOA. (**G**) The molecule interaction between ALDOA. (**H**) The molecules selected between ALDOA and FBP1 are increased in LIHC. N is denoted the normal sample size. T is denoted as the tumor sample size. HR is denoted as hazard ratio. The significance of the differences was analyzed using the GEPIA website.
