# Supplementary material for: The role of fructose 1,6-bisphosphate-mediated glycolysis/gluconeogenesis genes in cancer prognosis
Source: Aging (Albany NY). 2022 Apr 11;14(7):3233–58. doi: 10.18632/aging.204010 (PMC9037270; doi:10.18632/aging.204010)
Supplement: Supplementary Tables 7 and 8 [file aging-14-204010-s008.pdf]

SUPPLEMENTARY TABLES

Supplementary Table 7. Genes overlapped to ALDOA and FBP1 expression of LIHC and LUAD in the TCGA (PanCancer Atlas dataset and Firehose Legacy dataset).

Overlapped gene
AURKB
CCNB1
SHCBP1
MTFR2
RAD51
KIF4A
PPM1G
TACC3
KIF2C
BIRC5
PLK1
ORC6
UBE2C
CENPA
CDT1
FAM72B
ORC1
CDC20
CENPH
CTSV
RAD54L
CDC45
NCAPG
MYBL2
CDCA5
RAN
UBE2T
SLC2A1
MAD2L1
CDCA8

Supplementary Table 8. Overlapped upstream regulators in the LIHC and LUAD.

Overlapped gene
VDR
ARNT
IRF4
YY1
TCF3
TP53
MED1
CEBPD
E2F5
FOXO3
MITF
HMGN5
CBX3
RRP1B
KLF5
CDKN2A
TBX2
CEBPB
HSF2
MYC
TAL1
MAX
SMAD7
E2F1
E2F7
BRCA1
E2F3
CBX4
MBD2
FOXO1
NUPR1
HIF1A
TP73
TLX1
MYBL2
ATF3
TCF4
ID2
SPI1
HDAC1
YAP1
RB1
TP63
RBL1
VHL
HSF1
NFYA
SP1

ZBTB17
CCND1
FOXM1
MLIP
NFE2L2
E2F4
GON4L
MYOD1
E2F2
